# An Exception Handling Approach for Privacy-Preserving Service Recommendation Failure in a Cloud Environment

**DOI:** 10.3390/s18072037

**Published:** 2018-06-26

**Authors:** Lianyong Qi, Shunmei Meng, Xuyun Zhang, Ruili Wang, Xiaolong Xu, Zhili Zhou, Wanchun Dou

**Affiliations:** 1 School of Information Science and Engineering, Qufu Normal University, Rizhao 276826, China; 2 School of Computer Science and Technology, Nanjing University of Science and Technology, Nanjing 210094, China; mengshunmei@njust.edu.cn; 3 State Key Laboratory for Novel Software Technology, Department of Computer Science and Technology, Nanjing University, Nanjing 210023, China; douwc@nju.edu.cn; 4 Department of Electrical and Computer Engineering, University of Auckland, Auckland 1023, New Zealand; xuyun.zhang@auckland.ac.nz; 5 Institute of Natural and Mathematical Sciences, Massey University, Auckland 0745, New Zealand; Ruili.WANG@MASSEY.AC.NZ; 6 School of Computer and Software, Jiangsu Engineering Centre of Network Monitoring, Nanjing University of Information Science and Technology, Nanjing 210044, China; xlxu@nuist.edu.cn (X.X.); zhou_zhili@163.com (Z.Z.)

**Keywords:** service recommendation, privacy-preservation, failure, exception handling, converse Locality-Sensitive Hashing

## Abstract

Service recommendation has become an effective way to quickly extract insightful information from massive data. However, in the cloud environment, the quality of service (QoS) data used to make recommendation decisions are often monitored by distributed sensors and stored in different cloud platforms. In this situation, integrating these distributed data (monitored by remote sensors) across different platforms while guaranteeing user privacy is an important but challenging task, for the successful service recommendation in the cloud environment. Locality-Sensitive Hashing (LSH) is a promising way to achieve the abovementioned data integration and privacy-preservation goals, while current LSH-based recommendation studies seldom consider the possible recommendation failures and hence reduce the robustness of recommender systems significantly. In view of this challenge, we develop a new LSH variant, named converse LSH, and then suggest an exception handling approach for recommendation failures based on the converse LSH technique. Finally, we conduct several simulated experiments based on the well-known dataset, i.e., Movielens to prove the effectiveness and efficiency of our approach.

## 1. Introduction

With the advent of Web of Things (WoT), an increasing number of enterprises or organizations are apt to encapsulate their products (e.g., web API (Application Programming Interface)) into easy-to-access web services and publish them on the web so as to attract potential users and gain more profits. However, the ever-increasing volume and varieties of candidate services place a heavy burden on the service selection decisions of target users [1]. Under the circumstance, Collaborative Filtering (CF)-based recommendation techniques are proposed to minimize such burdens. Typically, for a target user who requires recommend services, the recommender system can look for his/her similar friends by observing the quality of service (QoS) data monitored by various sensors, and then enact appropriate recommendation decisions with the help of derived friends. Nowadays, CF technique has been successfully applied in many recommender systems whose decision-making data for recommendation are organized or stored in a centralized way.

However, in the cloud computing environment, the QoS information that is crucial to recommendation decisions is often not centralized, but rather monitored by distributed sensors and stored in different cloud platforms [2]. In this situation, it is necessary for a recommender system to integrate or fuse these distributed data across different cloud platforms quickly and properly, so as to make comprehensive and accurate recommendation decisions. In particular, to protect the sensitive business information and obey the laws [3,4,5], preserving user privacy during the abovementioned multi-source data integration process is an important but challenging task [6,7,8,9,10] for the success of subsequent recommendations.

The Locality-Sensitive Hashing (LSH) technique [11] has recently been recruited to make efficient and privacy-preserving service recommendation in the distributed environment. Typically, according to the QoS data, the Locality-Sensitive Hashing technique can be used to search for the similar friends of a target user in an efficient and privacy-preserving manner. Afterwards, recommended results are generated by considering the preferences of obtained similar friends. However, in certain situations, the recommender system cannot generate or produce any satisfying recommended result; in other words, a recommendation failure occurs. While existing LSH-based service recommendation approaches seldom consider this kind of recommendation failure problems as well as the corresponding exception handling solutions; therefore, the robustness of the recommender system is reduced significantly.

**Figure 1:**
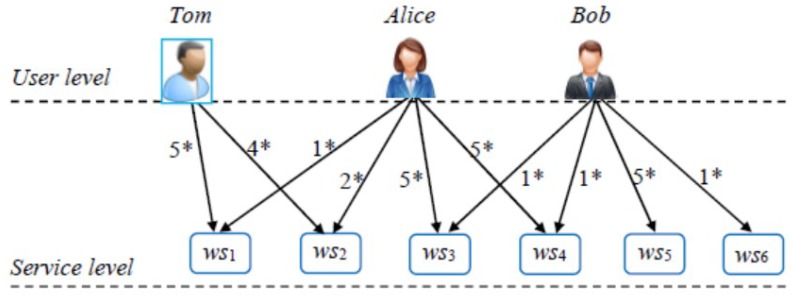
A recommendation failure example (see our previous work [12]).

An intuitive example is presented in Figure 1, which contains three users and six services. The user ratings are denoted by 1*–5*. According to the traditional LSH technique, the index values of *Tom* and *Alice* are not same as they have no co-invoked services. Therefore, *Tom* is not similar with *Alice*. Likewise, as Figure 1 shows, *Tom* is not similar with *Bob* either. In this situation, no satisfying candidate services can be recommended or returned to *Tom*, i.e., the service recommendation process is failed.

In view of this challenge, we propose converse LSH technique and utilize it to look for a target user’s contrary users (denoted by “enemy” in this paper) whose preferences are totally different from the target user. Afterwards, according to the enemies of the target user, we infer the possible friends of the target user indirectly so as to handle the exception incurred by recommendation failures. Overall, the contributions in this paper are as follows:

(1) A novel LSH variant named converse LSH is developed, which can be utilized to search for the enemy users of a target user, in a time-efficient and privacy-preserving way.
(2) We utilize converse LSH technique to search for the enemies of a target user and then look for the target user’s similar friends indirectly based on the “enemy’s enemy is a possible friend” inference rule in Social Balance Theory. Afterwards, we generate recommended results by considering the preferences of obtained similar friends, so as to handle the exceptions incurred by recommendation failures.
(3) Comprehensive experiments are simulated based on Movielens dataset, to test the effectiveness of suggested recommendation approach. Experiment results indicate the advantages of our proposal compared to other competitive approaches when a recommendation failure occurs.

The rest of this paper is structured as follows: in Section 2, we introduce the related work. Converse LSH technique is proposed in Section 3. An exception handling approach based on converse LSH is put forward in Section 4, to achieve indirect friend finding and service recommendations. Experiment evaluations are given in Section 5. In Section 6, we summarize the paper and point out the future research directions.

## 2. Related Work

Many researchers have investigated the privacy concerns in recommendation process and provide their respective resolutions. In [13], the authors suggested that a user can publish partial QoS data to the service community, so as to protect the remaining majority of QoS data. Similarly, in [14], the authors take the amount of published data as a tunable parameter and then transform the privacy-preservation problem into a multi-object optimization problem, so as to achieve a good tradeoff between data availability and data privacy. However, in the above approaches, certain sensitive information about users may be in danger due to the published partial data. Besides, recommendation failures are not considered in these approaches.

Microaggregation idea is adopted in [15] to realize data K-anonymization so that the users’ sensitive data (e.g., user location) can be protected. However, there is often a tradeoff between data availability and data privacy; so the recommendation accuracy is often not as high as expected if the anonymous data are employed to make service recommendation decisions. Besides, these approaches do not discuss the possible service recommendation failures. Encryption technique is adopted in work [16] to guarantee the privacy-preservation of sensitive information. However, as a heavy-weight privacy-preservation manner, encryption operations often lead to high computational cost and long delay; therefore, the encryption techniques are often not applicable to the light-weight service recommendation requirements from certain users. Besides, recommendation failures are out of the scope of these encryption-based approaches. 

Randomized disturbance idea is adopted in [17] to convert the real QoS data into the disturbed data; afterwards, the latter data are regarded as the recommendation bases to achieve the privacy-preservation goal. However, recommendation failures are not discussed; besides, the applicability of this approach is relatively limited as it can only be applied to the Pearson Correlation Coefficient (PCC)-based collaborative service recommendation scenarios. In [18], the authors utilize the Differential Privacy technique to make noise data injection and confusion, so as to ensure that the real service quality data would not be exposed to the outside. However, the time complexity of Differential Privacy is relatively high; second, when the service quality data are updated frequently, the accumulated noise would be enlarged, which will decrease the service recommendation accuracy accordingly; third, they do not consider the recommendation failures.

As an effective and efficient way to search for similar friends in the big data context, LSH is recently introduced into service recommendation to achieve the distributed data integration and privacy-preservation goals. In our previous work [19,20,21], LSH is combined with user-based CF to make privacy-preserving service recommendation. Likewise, in [22], LSH is combined with item-based CF to build service index table with little privacy and then make service recommendation based on the service index table. However, these LSH-based recommendation approaches do not consider the recommendation failures as well as the corresponding exception handling resolutions. As to the recommendation failures, the authors in [23] adopt the average idea to predict the missing QoS data. Social Balance Theory is utilized in our previous works [12,24] to look for the possible friends of a target user so as to cope with the recommendation failures. However, privacy concerns are not discussed.

Through the above literature review, a conclusion can be drawn that existing recommendation approaches either fail to protect user privacy or overlook the recommendation failures and exceptions. In view of this drawback, we propose converse LSH technique and utilize it to handle the exceptions incurred by recommendation failures; this way, the robustness of recommender systems can be improved significantly.

## 3. Converse Locality-Sensitive Hashing

Traditional LSH is an effective similar neighbor search technique. Therefore, in the service recommendation domain, LSH is often integrated with user-based Collaborative Filtering (CF) technique to search for the similar friends of a target user, in an efficient and privacy-preserving way. In this section, we modify the traditional LSH technique and transform it into converse LSH which can be used to search for the enemies of a target user efficiently while guaranteeing user privacy. Next, we introduce the rationale of converse LSH. 

Let’s consider two *n*-dimensional vectors *X* = (*x*_1_, …, *x_n_*) and *Y* = (*y*_1_, …, *y_n_*) whose similarity can be depicted by the PCC distance. Next, according to LSH theory [11], we can transform vectors *X* and *Y* containing private information into corresponding hash values with little privacy, i.e., *h*(*X*) and *h*(*Y*), respectively. Concretely, *h*(*X*) can be calculated by (1), where *V* is an *n*-dimensional vector (*v*_1_, …, *v_n_*) and *v_j_* (*j* = 1, 2, … *n*) is randomly selected from [−1, 1]; “°” represents the inner product of different vectors. The physical meaning of equation in (1) is: vector *V* is a hyper plane which divides the *n*-dimensional space into two parts; if point *X* is above hyper plane *V* (i.e., *X*○*V* > 0), then *h*(*X*) = 1 with high probability; otherwise, *h*(*X*) = 0:

(1) $$h(X)=\{\begin{array}{ll} 1 & \mathrm{if}\;X○V>0\\ 0 & \mathrm{if}\;X○V\leq 0 \end{array}$$

Thus, through the hash map in (1), *n*-dimensional vectors *X* and *Y* are transformed into two Boolean values, i.e., *h*(*X*) and *h*(*Y*), respectively. However, LSH is essentially a probability-based similar friend search technique; therefore, a single hash value *h*(*X*) or *h*(*Y*) cannot precisely represents the original *n*-dimensional vector *X* or *Y*. Considering this, more hash functions, i.e., a hash function family *H*(.) = {*h*_1_(.), …, *h_r_*(.)} (*r* << *n*) are adopted here. Through the hash function family *H*(.), we can transform the *n*-dimensional vectors *X* and *Y* into *r*-dimensional 0–1 vectors, i.e., *H*(*X*) = {*h*_1_(*X*), …, *h_r_*(*X*)} and *H*(*Y*) = {*h*_1_(*Y*), …, *h_r_*(*Y*)}, respectively. The vectors *X* and *Y*, as well as their respective hash values *H*(*X*) and *H*(*Y*), form a hash table. We repeat the above hash table building process until *L* hash tables, i.e., *Tb*_1_, …, *Tb_L_* are obtained. Next, according to LSH theory, vectors *X* and *Y* are contrary with large probability iff the condition in (2) holds. In (2), *H_z_*(*X*) and *H_z_*(*Y*) denote the hash values of vectors *X* and *Y* in *z*-th hash table, respectively; symbol “⊕” represents the XOR operation:

∃ *z*, s.t. *H_z_*(*X*)⊕*H_z_*(*Y*) = (1, 1, …, 1) (*z* ∈ {1, …, *L*}) (2)

The physical meaning of equation in (2) is clarified as below: if points *X* and *Y* are always located on the different sides of hyper plane *V* (i.e., *h_i_*(*X*) ≠ *h_i_*(*Y*) holds for all *i* ∈ {1, ..., *r*}), then *X* and *Y* are far away from each other with large probability (i.e., *H*(*X*)⊕*H*(*Y*) = (1, 1, …, 1)). Furthermore, if *H*(*X*)⊕*H*(*Y*) = (1, 1, …, 1) in any of *Tb*_1_, …, *Tb_L_*, points *X* and *Y* can be regarded as two contrary points (i.e., enemies). This is the main idea of our proposed converse LSH technique. Through converse LSH, we can search for the users (denoted by “enemies”) whose preferences are totally different from the target user, in an efficient and privacy-preserving manner, as elaborated in the next section.

## 4. An Exception Handling Approach Based on Converse LSH

Next, we introduce an approach for handling the exceptions incurred by service recommendation failures, named SerRec_converse-LSH_, based on the converse LSH technique introduced in Section 3. Concretely, our approach consists of three steps.

- Step-1: Build user indices offline through traditional LSH technique.

Let’s consider a user *u* whose single hash value (denoted by *h*(*u*)) is based on the hash map in Equation (1) and user *u*’s historical service quality data (assume fixed and real values) monitored by sensors. Furthermore, according to the hash function family *H*(.) = {*h*_1_(.), …, *h_r_*(.)} (*r* << *n*) in Section 3, we can obtain user *u*’s compound hash value *H*(*u*) = {*h*_1_(*u*), …, *h_r_*(*u*)}. Then *H*(*u*) is treated as user *u*’s index. Moreover, all users as well as their respective indices form a hash table. Repeat the above process until *L* hash tables, i.e., *Tb*_1_, …, *Tb_L_* are obtained. The above user indices building process can be executed offline before a service recommendation requirement is raised; therefore, its time complexity is O(1), which indicates that the recommendation speed can be accelerated greatly.

- Step-2: Determine the indirect friends of the target user u* based on user indices and converse LSH technique.

We have obtained the user indices *H*(*u*) (including the index *H*(u*) for the target user (denoted by u*)), and form *L* hash tables *Tb*_1_, …, *Tb_L_*. Next, if *H*(*u*) ⊕ *H*(u*) = (1, 1, …, 1) holds in any *Tb*_1_, …, *Tb_L_*, then user *u* can be regarded as a qualified enemy of the target user u* based on the converse LSH theory introduced in Section 3. Likewise, for each user *ω* (*ω* ≠ *u* and *ω* ≠ u*), if *H*(*ω*) ⊕ *H*(*u*) = (1, 1, …, 1) holds in any hash table, then user *ω* can be regarded as a qualified enemy of user *u*. Thus, *ω* can be considered as an indirect friend of u* based on the “enemy’s enemy is a possible friend” rule in Social Balance Theory; afterwards, we put *ω* into a new user set Possible_Friend_set(u*). Repeat the above process until all the indirect friends of u* are found. This way, we can derive the friends of u* in an indirect manner, if u* does not have similar friends due to the data sparsity according to the traditional LSH technique. 

**Figure 2:**
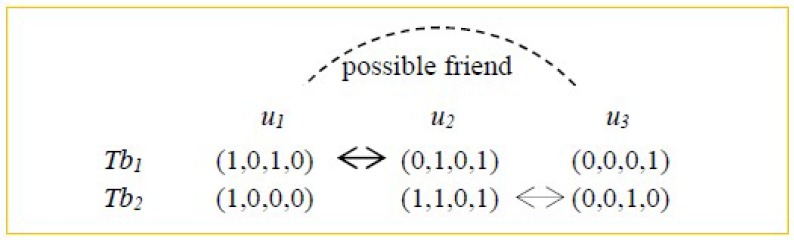
Indirect friend finding based on converse LSH: an example.

Next, we turn to the example in Figure 2 where three users {*u*_1_, *u*_2_, *u*_3_} and two hash tables {*Tb*_1_, *Tb*_2_} are present. The index values of the three users are also shown in Figure 2. Then according to the judgement condition in Equation (2), *u*_2_ is an enemy of *u*_1_ as (1, 0, 1, 0) ⊕ (0, 1, 0, 1) = (1, 1, 1, 1) holds in hash table *Tb*_1_. Similarly, *u*_3_ is an enemy of *u*_2_ as (1, 1, 0, 1) ⊕ (0, 0, 1, 0) = (1, 1, 1, 1) holds in hash table *Tb*_2_. With the above analyses, we can infer that *u*_3_ is a possible friend of *u*_1_ based on the “enemy’s enemy is a possible friend” rule. So *u*_3_ is put into the friend set of *u*_1_, i.e., Possible_Friend_set(u_1_).

- Step-3: Recommend services to u* based on the possible friends of u*.

According to target user u*’s possible friends (Possible_Friend_set(u*)) derived in Step-2, the users in, we can recommend appropriate services to u*. First of all, we predict the missing QoS data of *ws* by u* by (3) where *q* is a QoS criterion, *q*(u*, *ws*) represents *ws*’s QoS over criterion *q* invoked by u*; *Ф* denotes the set of users who are possible friends of u* and have invoked service *ws* before, which can be obtained by (4). Thus we can rank services *ws* based on *q*(u*, *ws*) in (3). At last, optimal services are recommended to u*; this way, the recommendation failures are overcome:

(3) $$q(u^{*},ws)={\frac{1}{\left|\varphi \right|}}^{*}\sum_{u_{i}\in \varphi }q(u_{i},ws)$$

*Ф* = {*u_i_*|*u_i_* ∈ Possible_Friend_set(u*) and *u_i_* has ever invoked *ws*} (4)

## 5. Experiments

### 5.1. Experiment Configurations

To prove the effectiveness of our suggested exception handling approach named SerRec_converse-LSH_, we design and deploy several experiments based on popular dataset Movielens [25]. Movielens reports the rating data of 3900 movies rated by 6040 users all over the world. Different from other applications where multiple dimensions are present [26,27,28,29,30,31,32,33,34], we consider only one quality dimension (i.e., user rating) in the experiments and take it as the unique recommendation basis, because the Movielens dataset only provides one dimension, i.e., user-service rating. The complex multi-dimensional service recommendation scenarios are out of the scope of this work. (In the multi-dimensional service recommendation scenarios, the multiple quality dimensions as well as their mutual correlations, such as the linear correlations and non-linear correlations should all be taken into consideration. In this situation, the problem becomes more complex and cannot be directly extended from this work, so we will investigate the complex multi-dimensional service recommendation problems in the future; see Section 5.3 “Shortcoming analyses & future work”). We randomly remove partial entries of the dataset to simulate recommendation failure scenarios. To evaluate the performance of exception handling approaches, we test the time cost and Mean Absolute Error (MAE), respectively (Note that LSH can protect data inherently, therefore, the privacy protection effect of SerRec_converse-LSH_ is not tested in the experiment). Moreover, to validate the feasibility of our proposed SerRec_converse-LSH_ approach, we compare our proposal with the following three competitive handling approaches.

(1) Random: this benchmark approach predicts the missing service quality data based on the quality of a randomly selected service, and returns the service with the optimal predicted quality.
(2) WSRec [23]: it predicts the missing service quality data by two pieces of average quality, i.e., average quality of the service rated by all users and average quality of all services rated by the user. Finally, the optimal service is returned to the target user.
(3) SBT-SR [12]: this approach first looks for the indirect friends of a target user based on Collaborative Filtering and Social Balance Theory, and then recommends appropriate services based on the derived indirect friends.

The experiment running environment is as follows: (1) hardware: 2.80 GHz CPU + 2.0 GB RAM; (2) software: Windows XP + JAVA 1.5. Experiments are executed ten times and their average values are reported.

### 5.2. Experiment Results

Four experiments are designed and deployed, respectively. Four parameters are present in the experiments: *m*, *n* denote the sizes of user set and service set; *L*, *r* represent the sizes of hash table set and hash function set.

- Profile 1: Accuracy comparison of four approaches

**Figure 3:**
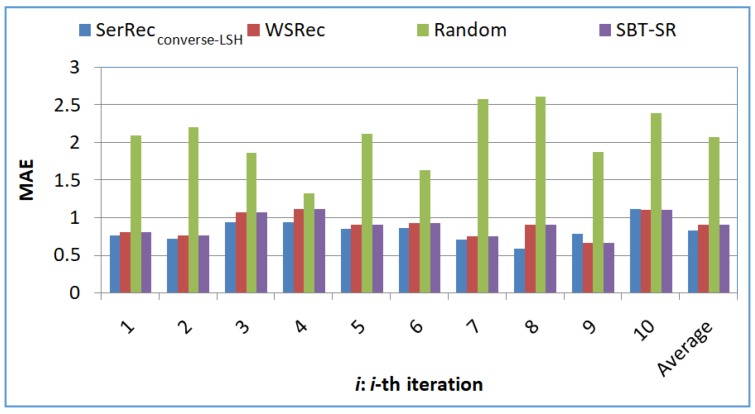
Accuracy of recommended results.

The accuracy values of outputted results of four exception handling approaches are compared. Here, *m* = 6000, *n* = 3900, *L* = 10, *r* = 8. Experiments are repeated ten times. The concrete experiment results of the ten iterations and the average result are demonstrated in Figure 3, which shows that the accuracy value of Random approach is the smallest, as a random strategy is adopted to predict the missing quality data. A simple and naïve “average” strategy is recruited in WSRec approach to predict the missing service quality, while the average service quality cannot reflect the real running quality of services very well; therefore, the recommendation accuracy of WSRec approach is also low. Both SerRec_converse-LSH_ and SBT-SR approaches utilize the Social Balance Theory to improve the recommendation robustness; however, the accuracy value of our suggested SerRec_converse-LSH_ approach outperforms those of the other three competitive approaches including SBT-SR, as only the similar friends (obtained in an indirect manner) of a target user are taken into consideration in missing QoS prediction in SerRec_converse-LSH_. Another observation from Figure 3 is that the recommendation accuracy value of SerRec_converse-LSH_ approach does not vary significantly and regularly, which means that our proposal can make relatively stable service recommendations.

- Profile 2: Efficiency comparison of four approaches

**Figure 4:**
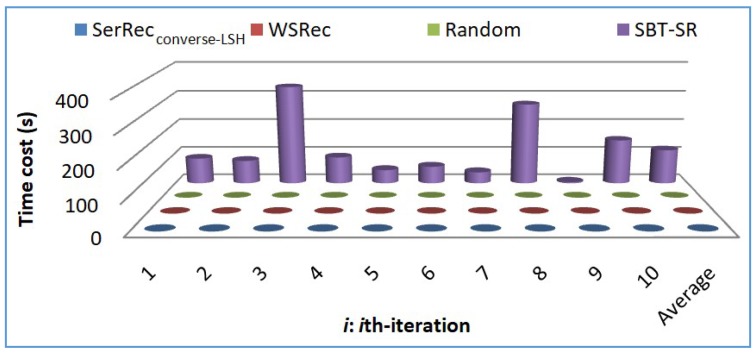
Recommendation efficiency comparison.

We measure the time cost for generating recommended results in our suggested SerRec_converse-LSH_ approach and compare it with the rest three approaches. The parameters are as follows: *m* = 6000, *n* = 3900, *L* = 10, *r* = 8. Experiments are repeated ten times. The concrete experiment results of the ten iterations and the average result are demonstrated in Figure 4.

Figure 4 shows that the recommendation efficiency of SBT-SR is low as it is based on Collaborative Filtering and hence the time cost is rather high. The service recommendation efficiencies of the other three approaches, i.e., SerRec_converse-LSH_, WSRec and Random are rather high and approximately the same. This is because most tasks in our SerRec_converse-LSH_ approach can be done offline, and both WSRec and Random approaches have a polynomial time complexity.

- Profile 3: Accuracy of SerRec_converse-LSH_ with respect to *L* and *r*

**Figure 5:**
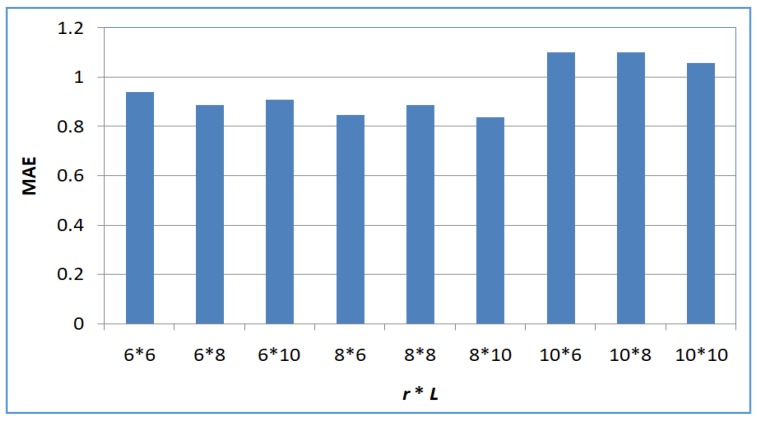
Accuracy of SerRec_converse-LSH_.

Next, we test the variation tendency of accuracy of the proposed SerRec_converse-LSH_ approach with respect to the parameters *L* and *r*. Here, *m* = 6000, *n* = 3900, *L* and *r* are both varied from 6 to 10. Experiments are repeated ten times. The average values are demonstrated in Figure 5.

According to LSH theory, a larger *r* value or a smaller *L* value implies tighter condition for neighbor search and higher recommendation accuracy (i.e., lower MAE value). However, as Figure 5 indicates, the recommendation accuracy of SerRec_converse-LSH_ does not render an obvious fluctuation tendency with *L* and *r*. This is due to the following reason: in our proposal, the traditional LSH technique is modified to be the converse LSH technique; and the converse LSH technique is recruited twice in order to search for the friends of a target user indirectly. So the influence of parameters *L* and *r* over the recommendation accuracy is not so obvious any more.

- Profile 4: Efficiency of SerRec_converse-LSH_ with respect to *L* and *r*

**Figure 6:**
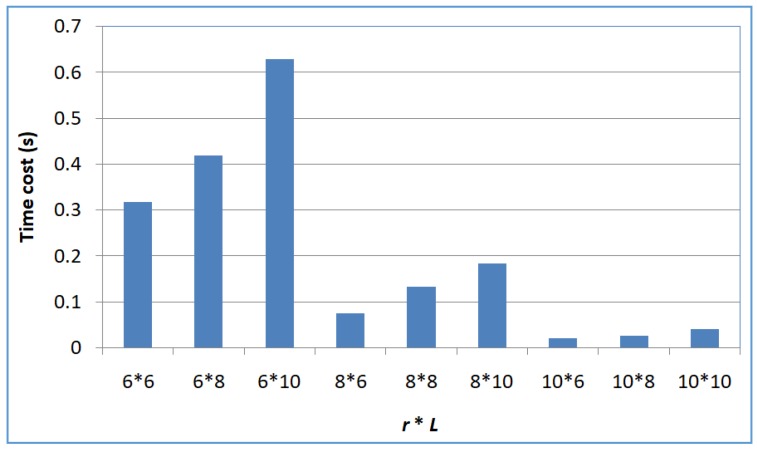
Efficiency of SerRec_converse-LSH_.

Next, we evaluate the efficiency of SerRec_converse-LSH_ with respect to *L* and *r*. Here, *m* = 6000; *n* = 3900; *L* = 6, 8, 10; *r* = 6, 8, 10. Experiments are repeated ten times. The average results are demonstrated in Figure 6.

From the figure, we can see that our efficiency decreases when the number of hash tables, i.e., *L* rises. This is because when *L* grows, the search condition for dissimilar enemy becomes looser and correspondingly, more qualified enemies are returned to take part in the service recommendation decision-makings; in this situation, more time cost is needed. Another result that Figure 6 indicates is that our efficiency decreases when the number of hash functions, i.e., *r* drops. This is because when *r* drops, the search condition for dissimilar enemy becomes stricter and correspondingly, fewer qualified enemies of a target user are returned to take part in the service recommendation decision-makings; therefore, less computational time is needed.

With the above analyses, a conclusion can be drawn that SerRec_converse-LSH_ approach achieves a good tradeoff between service recommendation accuracy and efficiency. Besides, SerRec_converse-LSH_ outperforms the other approaches in terms of privacy-preservation due to the inherent characteristic of LSH.

### 5.3. Shortcoming Analyses & Future Work

There are still several shortcomings in our approach. First of all, we only consider the recommendation scenario where one quality dimension is monitored by sensors, while multi-dimensional and weighted applications are more common in practice [35,36,37], so in the future, we will further refine our work by considering the multiple service quality dimensions as well as their respective weights. Besides, for simplicity, we only discuss the service quality dimensions with real and continuous monitored values, without considering the diversity of the quality values (e.g., discrete [38,39], binary [40], fuzzy [41] and correlated [42,43,44]). Considering this drawback, we will further improve our proposed recommendation approach by integrating the diverse forms (or formats) of different service quality dimensions, for the purpose of getting more comprehensive and reasonable recommended services.

## 6. Conclusions

The multi-source property of service usage data (monitored by distributed sensors) used to make service recommendations in the cloud environment requires that a recommender system to quickly integrate the distributed monitored data so as to make comprehensive and accurate recommendation decisions. In this situation, protecting the private information of users from leakage during the above data integration process is an important but challenging task for the successful service recommendation. Although the LSH technique can be recruited to achieve the abovementioned data integration and privacy-preservation goals, existing LSH-based service recommendation approaches seldom consider the possible recommendation failures as well as the resulted exceptions. In view of this drawback, we put forward a new LSH variant, i.e., converse LSH, and integrate it with the Social Balance Theory so as to look for the possible friends of a target user indirectly and then recommend appropriate services based on the obtained possible friends. The experiments conducted on Movielens dataset prove the effectiveness of our approach in terms of service recommendation accuracy and time cost while guaranteeing privacy-preservation of quality data monitored by sensors.

However, in SerRec_converse-LSH_, only one quality dimension of services is considered. In our future work, we will continue to refine SerRec_converse-LSH_ by considering multiple quality dimensions as well as their weight information. Besides, QoS data often vary with concrete service execution context (e.g., service invocation time and user location); therefore, we will further improve our approach by taking context into consideration.
